# An application of a series of theory-based educational intervention based on the health belief model on skin cancer prevention behaviors in female high school students

**DOI:** 10.1016/j.heliyon.2023.e17209

**Published:** 2023-06-18

**Authors:** Amirhossein Kamyab, Tahereh Gholami, Kasra Behdad, Ali Khani Jeihooni

**Affiliations:** a Faculty of Medicine, Fasa University of Medical Sciences, Fasa, Iran; b Department of Public Health, School of Health, Fasa University of Medical Sciences, Fasa, Iran; c Department of Dermatology, School of Medicine, Fasa University of Medical Sciences, Fasa, Iran; d Nutrition Research Center, Department of Public Health, School of Health, Shiraz University of Medical Sciences, Shiraz, Iran

**Keywords:** Students, Intention, Behavior, Prevention and control, Skin neoplasms, Knowledge, Attitude

## Abstract

**Background:**

Cancer is a leading cause of death globally and the second cause of death in developed countries. Having a rising incidence, skin cancer is the most prevalent cancer in Iran. Long-term UV radiations, particularly during childhood and adolescence, is a major cause of skin cancers. The Theory of Planned Behavior as the most precise indicator of behavior, contains motivational factors affecting behavior. This theory has been successful in predicting factors related to chronic diseases, especially cancer. As this model was successful in assessing sun-protective behaviors in previous studies, this study was designed to figure out how a theory-based educational intervention can affect the skin cancer prevention practices of Iranian female high school students.

**Methods:**

This experimental investigation was carried out 2019–2020 using multistage cluster sampling method on 400 female high school students in Fasa, Fars, Iran. A questionnaire consisting of demographic data and a questionnaire including the components of the Theory of Planned Behavior was used to assess skin cancer preventative behaviors of both the trial and control groups of the study. An educational program based on the Theory of Planned Behavior was held for the experimental group for eight weeks regarding skin cancer preventive behaviors. The two groups completed questionnaires three months following the intervention for a second time.

**Results:**

The study's findings revealed that prior to the intervention, there was no considerable distinction between the two study groups with regard to knowledge, attitude, subjective norms, perceived behavioral control, behavioral intentions, and skin cancer prevention behaviors; yet, three months later, the experimental group demonstrated increases in each of the mentioned variables with a significant difference. In contrast, the control group showed no discernible difference.

**Conclusions:**

The findings of this investigation highlighted the success of the Theory of Planned Behavior for designing educational interventions aimed at encouraging skin cancer prevention in a population of female high school students.

## 1 Introduction

Today, the growing number of cancer patients globally deems cancer as an international health concern and makes fighting it one of the health care priorities. Cancer is a known leading cause of death worldwide and the second most significant cause of death in prosperous nations [1]. The number of cancer patients has increased worldwide [2]. Following coronary artery disease and traffic incidents, cancer is the primary cause of death in Iran [3]. While, a great proportion of all types of cancers are preventable through preventive measures and avoiding the contributing factors [4].

Skin cancer has the highest frequency among malignancies among the white race [5]. In accordance with WHO statistics, 132 000 cases of melanoma and 2–3 million cases of non-melanoma skin cancers are diagnosed each year [6]. Earlier studies estimated the occurrence of various subtypes of skin cancers to 20 million by 2020 [7]. Sunlight exposure is responsible for 90% of all incidences of skin cancer [8]. Having a rising incidence, skin cancer has the highest prevalence among other types of cancer in Iran [9]. The elevated rate of skin cancer in Iran could be as a consequence of intense sunshine in the most times of the year and the absence of proper protective clothing or headwear among Iranians [10]. Skin cancer is more prevalent in southern regions of Iran compared to other parts of the country due to the higher UV radiations [9].

Children and adolescents are more in danger of developing skin malignancies, as they are more exposed to the sunlight, particularly on weekends and throughout the summer [11]. One of the WHO's initiatives in cancer control is to raise public knowledge towards cancer [12]. Yet, numerous studies have demonstrated that raising the knowledge about the risks of skin cancer had no long-term effects on skin cancer preventive behaviors in adolescents; therefore, they should be encouraged to improve their attitudes and behaviors regarding exposure to sunlight [13]. Hence, implementing educational interventions focusing on the promotion of behaviors for preventing skin cancer could have positive effects on this age group [14].

One of the most successful models of health promotion and education is the Theory of Planned Behavior (TPB) [15]. This theory was created by Ajzen and Fishbin in 1988 in the field of social psychology based on the cognitive-social and expectation-value models [16]. The components of this theory include attitude, subjective norms, perceived behavioral control, behavioral intention, and behavior [16]. TPB is the most precise indicator of behavior, containing motivating factors affecting behavior. In this theory, intention is introduced as a necessary and immediate predictor for behavior; in other terms, the more powerful the intentions, the more success is expected in adopting a behavior [15]. Many researchers have used this theory as a guidance in conducting their studies. A review of the literature shows the effectiveness of the TPB in anticipating factors related to chronic illnesses, especially cancers [17,18].

Since this model has been used in predicting human behavior in the prevention of disease, when utilized in interventions, it can alter the course of the diseases [19]. This model has been successful in conducting educational interventions regarding cancer prevention [20,21]. Previous studies used the TPB to evaluate sun protective behaviors; however, few used this model to implement an educational intervention promoting skin cancer preventive measures [22,23]. Considering the TPB's success in health education, the necessity of implementing an educational program regarding skin cancer prevention among adolescents, the high prevalence of skin cancer in southern areas of Iran, and the lack of proper education regarding this disease in Iran, this descriptive, cross-sectional study was designed to evaluate the impact of an educational program based on the TPB on skin cancer preventive practices in a population of female high school students in one of the southern cities of Iran, with high prevalence of skin cancer [24].

## 2 Materials and methods

### 2.1 Study design and population

In this experimental study, 400 female high school pupils from Fasa City, Fars Province, Iran, were involved in the 2019–2020 academic year. According to former investigations, a sample size of 400 individuals was determined with 95% confidence interval and 80% text power (200 people for the experimental group and 200 people for the control group) [25].

The participants were chosen through the multistage cluster sampling method. In this way, out of 26 public high schools for girls in Fasa, four were randomly chosen for the study. All public high schools in Fasa contain students from different social classes and are randomly scattered throughout the city. Two of the selected four high schools were chosen at random for each study group. Then, using computer-generated random number sequence, 100 individuals meeting the inclusion criteria were chosen from each high school (200 students for the experimental group and 200 for the control group).

The criteria for entering the study included being between 15 and 18 years old, studying in public high schools, and giving consent to take part in the research. The exclusion criteria included missing more than two educational sessions and providing incomplete answers to the questions.

From these four schools, two were selected randomly for the experimental group and two for the control group.

**Fig. 1:**
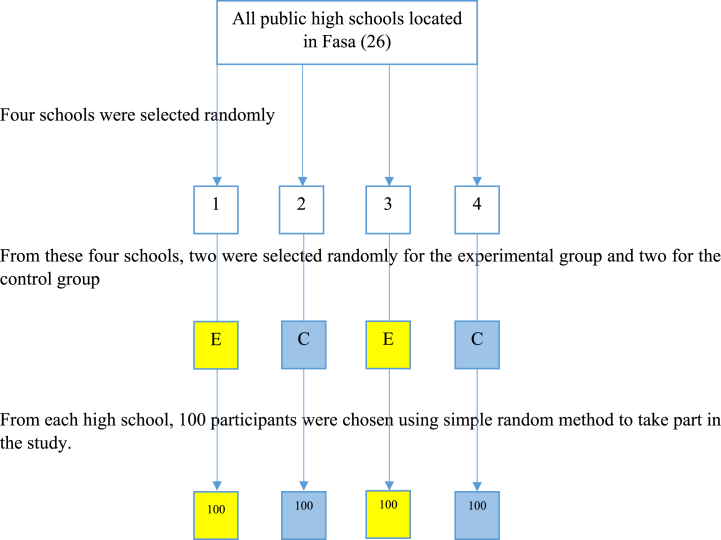
Flow chart of study.

From each high school, 100 participants were chosen using simple random method to take part in the study (Fig. 1).

### 2.2 Ethical considerations

Following the Declaration of Helsinki, this study was authorized by the Ethics Committee of The Fasa University Medical Sciences under the project number “IR.FUMS.REC.1399.029".

All students were made aware of the quality, data confidentiality policies, and the objectives of the study. Before completing the questions, participants selected the “I agree” choice to demonstrate their informed consent. In accordance with the Iranian ethics committee regulations, subjects were deemed emancipated minors. Thus, written informed consent was also obtained from the administrators of the care centers.

### 2.3 Questionnaires

The study's questionnaire was formed based on former studies data collection tools [[26], [27], [28], [29], [30], [31], [32]]. Initially, demographic information (age, monthly household income, father's education, mother's education, mother's occupation, father's occupation, and family history of skin cancer) were collected from the students. Student's knowledge of skin cancer was measured using 15 questions in the form of four options (the correct option scored one wrong options scored of zero), scoring from 0 to 15, a higher score indicated greater knowledge levels.

### 2.4 Theory of Planned Behavior questionnaire

Then, the TPB questionnaire was completed. This questionnaire, contained questions regarding students’ attitude, subjective norms, perceived behavioral control, behavioral intention, and skin cancer preventive behaviors. Attitude structure was evaluated using 12 questions on a 5-point Likert scale ranging from “completely agree” to “completely disagree,” with a score between 12 and 60. Subjective norms included 10 questions on a 5-point Likert scale (completely agree to completely disagree) and was scored between 10 and 50. Perceived behavioral control was assessed through 10 questions on a 5-point Likert scale (low probability to extremely high probability), with a minimum of 10 and a maximum of 50. The behavioral intention construct contained 15 questions on a 5-point Likert scale (from not at all true = 1 to completely true = 5), scoring from 15 to 75. Lastly, skin cancer preventive behaviors were also assessed using a scale of 15 questions (with yes/no responses), with a lowest value of zero and a top score of 15.

The items’ validity was assessed by determining an impact score index higher than 15.00 and a content validity ratio index greater than 0.79. In order to assess the face validity, a list of items was prepared based on 45 female high school students who shared similar demographic, economic, and social backgrounds as the target population. For assessing the validity of the questionnaire, the views of ten experts having health education and health promotion background and two dermatologists (outside the research team) were used.

According to Lawshe table, any item greater than the index (0.56 for 12 people), was considered significant and used for further analysis. The values estimated in this study were greater than 0.70 for most of the items. The overall reliability of the study tool was 0.88 by measuring Cronbach's alpha. The reliability of knowledge (0.80), attitude (0.89), subjective norms (0.88), perceived behavioral control (0.84), behavioral intention (0.85), and skin cancer prevention behaviors (0.88), was confirmed.

### 2.5 Procedure

After defining the study groups, the questionnaire was completed by the two study groups, then the instructional program was started for the experimental group. The training course consisted of eight training sessions for 50–55 min to weekly intervals of one session, and the topics and time were assigned in a WhatsApp group. The training sessions were planned by a doctorate in health education and health promotion, a dermatologist, and a senior occupational health expert. The program was based on the TPB and its educational topics, including the role of skin, the incidence rate of skin cancer and its contributing factors, the harms of sunlight, and how to protect from sunlight.

Educational videos, PowerPoint presentations, educational slides, and instructional booklets were used as educational aids. The sessions were conducted using lectures, question-and-answer sessions, small-group discussions, and role-playing methods. Some educational topics were discussed in the presence of teachers, school administrators, parents, and health care professionals. In addition, instructions on detecting dangerous situations, problem-solving skills, cognitive abilities, and the capacity to adopt new behaviors were included in the training sessions.

Once a week, an academic and motivational SMS was sent to the students through the WhatsApp group regarding skin cancer preventive measures. Moreover, the students received two monthly follow-up sessions. The two study groups answered the questionnaire once more three months following the intervention.

### 2.6 Data analysis

SPSS version 22 was used to analyze the data. The quantitative variables between the two groups were compared using the independent *t*-test, and comparing qualitative variables was performed using the chi-square test. Logistic regression test was applied for comparing behavior constructs between the control and experimental group, paired *t*-test for evaluating the effect of intervention on different parts of the TPB, and statistical significance was defined as p < 0.05.

## 3 Results

This survey included 400 female high school students in total. The average age was 16.1 + 1.59 years for the experimental group, and 15.97 + 1.70 years the control group. Based on the independent *t*-test, there was no significant difference regarding the ages of two groups (p = 0.318). As shown by the chi-square test, there was no statistically significant difference between the two groups' monthly household incomes (p = 0.234), fathers' educational levels (p = 0.216), mothers' educational levels (p = 0.244), fathers' occupations (p = 0.286), mothers' occupations (p = 0.366), and the family's history of skin cancer (p = 0.428) (Table 1).

**Table 1:** Comparison of the frequency distribution of demographic variables between the two study groups.

Variable		Experimental group N = 200		Control group N = 200		*P*-value
		Number	Percentage	Number	Percentage	
Monthly household income	Under 20 million Rod rials	73	36.50	65	32.50	0.234
	20 to 40 million rials	95	47.50	92	46	
	Over 40 million rials	32	16	43	21.50	
father's education level	Illiterate	3	1.5	2	1	0.216
	Elementary	32	18	28	14	
	Middle	42	21	40	20	
	High School	74	37	83	41.50	
	University	49	24.5	47	23.5	
Mother's education level	Illiterate	1	0.5	1	0.5	0.244
	Elementary	36	18	32	16	
	Middle	48	24	52	26	
	High School	83	41.5	78	39	
	University	32	16	37	18.5	
Father's occupation	Farmer	40	20	44	22	0.286
	Self-employment	67	34	56	28	
	Employee	58	29	62	31	
	Other items	34	17	38	19	
Mother's occupation	Housewife	157	78.5	161	80.5	0.366
	Employed	43	21.5	39	19.5	
Skin cancer history in the family	Have	13	6.5	10	5	0.428
	Do not have	187	93.5	190	95	

The finding revealed that prior to the intervention, there was no significant distinction comparing the two study groups in respect of knowledge, attitude, subjective norms, perceived behavioral control, behavioral intention, and skin cancer preventive behaviors (logistic regression, p > 0.05).

Nevertheless, three months following the intervention, the experimental group demonstrated improvements in knowledge (6.22 ± 1.52 to 12.26 ± 1.55), attitude (25.51 ± 5.48 to 52.36 ± 5.82), subjective norms (20.21 ± 5.20 to 43.32 ± 5.11), perceived behavioral control (17.64 ± 4.87 to 40.46 ± 4.58), behavioral intention (27.40 ± 5.33 to 61.46 ± 5.68), and in skin cancer preventive behaviors (5.13 ± 1.35 to 12.84 ± 1.30) with a significant difference (paired *t*-test, p < 0.05). In comparison, no noticeable distinction in was detected in the control group (Table 2).

**Table 2:** Comparison of average scores of the Theory of Planned Behavior constructs in the both study groups before and three months following the training intervention.

Variable	Groups	Before intervention M±SD	Three months after the intervention M±SD	*P*-value
Knowledge	Experimental	6.22 ± 1.52	12.26 ± 1.55	0.001
	Control	6.17 ± 1.67	7.03 ± 1.65	0.347
	p-value	0.318	0.001	
Attitude	Experimental	25.51 ± 5.48	52.36 ± 5.82	0.001
	Control	24.98 ± 5.55	25.79 ± 5.64	0.288
	p-value	0.299	0.001	
Subjective norms	Experimental	20.21 ± 5.20	43.32 ± 5.11	0.001
	Control	21.17 ± 5.02	22.62 ± 3.98	0.201
	p-value	0.285	0.001	
Perceived behavioral control	Experimental	17.64 ± 4.87	40.46 ± 4.58	0.001
	Control	19.10 ± 4.69	21.68 ± 4.70	0.147
	p-value	0.156	0.001	
Behavioral intention	Experimental	27.40 ± 5.33	61.46 ± 5.68	0.001
	Control	29.82 ± 5.41	32.73 ± 5.68	0.119
	p-value	0.142	0.001	
Skin cancer preventive behaviors	Experimental	5.13 ± 1.35	12.84 ± 1.30	0.001
	Control	5.28 ± 1.31	6.01 ± 1.14	0.284
	p-value	0.410	0.001	

## 4 Discussion

Adolescence is a developmental period of life during which significant behavioral patterns that have an impact on a person's entire life are established. During adolescence, people are influenced by family, friends, and the society. At this age, people lack the cognitive abilities necessary to evaluate different alternatives and predict the likelihood of their effects [33]. As a result, adolescence is a time to learn how to avoid preventable diseases such as skin cancer. According to a study by Wu and colleagues, a great majority of adolescents do not use enough sun protective strategies [34]. Finding at-risk groups and educating them about the early signs of skin cancer is one of the most crucial steps in controlling and preventing the disease. It is worth mentioning that encouraging people to change their attitudes and behaviors is a major key to control this disease.

Derive from our results, three months following the intervention, there was a notable improvement in the experimental group's average score of knowledge regarding skin cancer compared to the control group. WHO has identified strengthening people's knowledge as one of the main strategies for preventing malignancies. Holding educational sessions, displaying videos and PowerPoint presentations, question and answer sessions, and conducting small group discussions may have contributed to the growth in students' knowledge in this study. Consistently, in other similar studies, using visual learning methods has increased people's knowledge of skin cancer [31,[35], [36], [37]].

In this study, the instructional program increased the average score of people's attitudes toward skin cancer preventive practices in the experimental group in comparison with the control group. These findings are consistent with those of earlier investigations [26,[38], [39], [40]]. Since expressing ideas and seeing other people's reactions is one of the strongest ways to change attitudes, it is likely that in this study, using the role-playing method and encouraging people to express their opinions increased the participants' positive attitudes. Earlier studies have shown that the application of conventional and one-sided teaching methods and the passive participation of learners, do not have a significant effect on increasing the attitudes [41].

Following the intervention, the mean score of the subjective norms rose substantially in the experimental group in comparison with the control group. The involvement of teachers, parents, school administrators, and health care professionals in this study has increased subjective norms in the experimental group. In a study by Najafi et al. the most important influential people in the subjective norms of skin cancer preventive behaviors were parents, while the least perceived support was from their friends and relatives [42]. In another study by White et al. friends and peers positively affected the subjective norms of young girls toward sun protective behaviors [30].

In accordance with our findings, the experimental group's mean score for perceived behavioral control increased remarkably compared to the control group after the intervention. Perceived behavioral control reflects a person's perception of their ability to manage their behavior. When people believe they have control over their actions, they are more likely to engage with positive behaviors [43]. In this study, teaching how to identify risky situations, problem-solving skills, cognitive skills, and how to adopt new behaviors enhanced the perceived behavioral control in the experimental group. In comparable studies, the educational intervention improved the average score of perceived behavioral control in the studied subjects [39,40].

According to our results, the average score of behavioral intention and skin cancer preventive measures showed a sharp rise in the experimental group following the educational intervention. In the TPB, intention is the most significant factor in determining someone's behavior. Intentions include elements that motivate behavior and indicate how strongly people engage in the behavior and how strongly they strive for it [44]. In this study, education based on the TPB with an emphasis on appropriate encouraging and motivational feedback and engaging supporters as students' families, teachers, school administrators, and health care personnel, increased behavioral intentions and skin cancer prevention practices in students. Similar studies note that educational interventions increased intentions and skin cancer preventive behaviors, which support our findings [25,26,29,31,38,40].

Finally, according to the findings of this study, TPB was a suitable planning tool for an educational intervention aimed at enhancing adolescents' skin cancer preventive behaviors. The findings of other research support ours, highlighting the efficacy of TPB for creating effective educational intervention programs to enhance cancer prevention practices [20,21,45].

### 4.1 Limitations

One of the limitations of this study was that the intervention was exclusively performed on female students. It is necessary to conduct further research in this field on both male and female students simultaneously in order to compare the effect of education on gender differences. Moreover, this study was performed on a limited population, having a specific culture, since our findings could not represent the effect of education in the whole population. Further interventions on larger samples are recommended for more precise conclusions. Given the important role of teachers and parents in the students' education, similar trainings could be given to them for a better efficacy.

### 4.2 Strengths and weaknesses

One major strength of this study, was the implementation of the educational intervention based on the TPB, which has been known as one of the most effective models in generating cancer preventive behaviors.

Furthermore, the novel learning methods including, displaying instructional videos and pictures, question and answer sessions, small group discussions, the role-playing methods, involvement of students' acquaintances, and teaching cognitive skills, impressively enhanced skin cancer preventive behaviors in the participants of this study. Considering the importance of adolescence in adopting constructive lifelong behaviors, another strength of this study was the selection of the participants from this age group.

One of the weaknesses of this investigation was the self-reported data gathering, which may interfere with the exact assessment of the students' behavioral constructs. Further studies resting on the reviews of parents, friends, and teachers is recommended for enhancing the reliability of the data. Another weakness of this research was the lack of further follow-up investigations. Further follow-up investigations with lengthened periods are suggested to evaluate the long-term outcomes of the intervention.

## 5 Conclusion

The findings of the present study showed the success of the Theory of Planned Behavior in designing educational interventions regarding skin cancer prevention practices among female high school students.

### 5.1 Implications of the study

#### 5.1.1 Practice

Multipurpose interventions based on health education and promotion theories can be implemented in the early stages of adolescence to adopt preventive behaviors against skin cancer or other preventable types of cancer.

#### 5.1.2 Policy

School managers and health care policy makers can design stage-specific interventions in an interactive environment for students in their curriculum to facilitate preventive behaviors for them. These interventions and programs may include telephone consultations, use of social networks on mobile devices, participation in social activities and sports programs, and peer support.

#### 5.1.3 Research

Future studies can utilize health education and promotion theories in designing experimental interventions in various aspects of health, especially in the younger generation, to enhance the health levels in the future and reduce the burden of the health care system.

## Author contribution statement

Amirhossein Kamyab: Analyzed and interpreted the data; Contributed reagents, materials, analysis tools or data; Wrote the paper.

Tahereh Gholami: Performed the experiments; Analyzed and interpreted the data.

Kasra Behdad: Conceived and designed the experiments.

Ali Khani Jeihooni: Conceived and designed the experiments; Wrote the paper.

## Data availability statement

Data will be made available on request.

## Additional information

Supplementary content related to this article has been published online at [URL].

## Declaration of competing interest

The authors declare that they have no known competing financial interests or personal relationships that could have appeared to influence the work reported in this paper.

## Abbreviations

TPB: Theory of Planned Behavior
WHO: World Health Organization
