# Supplementary material for: An application of a series of theory-based educational intervention based on the health belief model on skin cancer prevention behaviors in female high school students
Source: Heliyon. 2023 Jun 18;9(6):e17209. doi: 10.1016/j.heliyon.2023.e17209 (PMC10333432; doi:10.1016/j.heliyon.2023.e17209)
Supplement: Multimedia component 1 [file mmc1.pdf]

Demographic Characteristics

1. Please write down your birth date (YYYY/MM/DD): _ _ _ _ / _ _ / _ _
2. Gender:
3. Are you a Spanish speaker?
   - Yes
   - No
4. How do you describe your skin color and race? (You may select more than one choice)
   - White/Caucasian
   - African American/Black
   - American Indian/Alaska Native
   - Asian/Pacific islands
   - Other (please specify):
5. Are you currently a student?
   - Yes
   - No
6. If not, what was your highest grade in school?
7. What is your current educational level?
   - Grade 5
   - Grade 6
   - Grade 7
   - Grade 8
   - Grade 9
   - Grade 10
   - Grade 11
   - Grade 12
   - Graduated from high school, but not currently studying
   - Graduated from high school, currently studying a two-year associate degree in a college
   - Graduated from high school, currently studying a four-year bachelor's degree in a college
8. During the last 12 months, how would you describe your grades in school out of 20?
   - More than 17
   - More than 15
   - More than 13
   - More than 11
   - Between 10 and 11
9. Are you currently employed?
   - Yes, working (full time)
   - Yes, part-time working and part-time student
   - No
10. Write the occupation and education level of your parents or your legal guardian:
    - Father:

- Mother:

11. Who do you live with? (Check all that apply)
    - With my mother
    - With my father
    - With my parents
    - With friends
    - With siblings, but not with parents
    - With other family members
    - With spouse/partner
    - With my children
    - With someone else (please specify):
    - I live alone
12. If you live in a dormitory, who do you live with at home?
13. Do you currently have health insurance?
    - Yes
    - No
14. What is the age of your family members?
    - Father:
    - Mother:
      Siblings:
    - Others:
15. How do you evaluate your current health status?
    - Excellent
    - Very well
    - Good
    - Moderate
    - Weak
16. How tall are you without shoes?
17. How much do you weigh without shoes?
18. Some people feel good by going to the doctor and getting a check-up despite being sick. How long has it been since the last time you went to the doctor?
    - Less than 1 year
    - 1-2 years ago
    - 3-4 years ago
    - 5 years or more
    - Never
19. If you went to a doctor, which of the following were among the recommendations of the doctor (if possible, specify which doctor's specialization):
    - Exercise more (how many times?)
    - Encouraging to regular mild exercises
    - Reduction of physical activities
    - Refraining from lifting weights
    - Smoking cessation

- Reducing/avoiding alcohol consumption
- Avoiding high-fat diet
- Weight loss
- Eating more fruits and vegetables
- Reducing sunlight exposure
- Preventing tanning
- Personal protection from the sunlight (for example using sunscreen creams, long sleeves, hats, etc.)
- Annual physical examination by an internist
- Frequent physical examinations by an internist
- Physical examinations, especially for cancer screening
- Skin checkups and examinations by yourself
- The doctor performed a skin examination
- Recommendation to take vitamins
- Other recommendations (specify):

Knowledge Questionnaire

People have different views about the possibility of getting cancer or something similar. Please mark your opinion about this in the following comments.

1. Please compare your chances of diagnosing cancer or similar diseases with others according to your gender and age. Compared to other people of my age and gender, my chances of being diagnosed with cancer or similar diseases (any type of cancer and similar diseases) are:
   - Much less than other people of my age and gender
   - A little less than other people of my age and gender
   - Almost like other people of my age and gender
   - A little more than other people of my age and gender
   - Much more than other people of my age and gender

2. Please compare the chances of heart diseases with others according to your age and sex. Compared to other people, my chances of having heart diseases are:
   - Much less than other people of my age and gender
   - A little less than other people of my age and gender
   - Almost like other people of my age and gender
   - A little more than other people of my age and gender
   - Much more than other people of my age and gender

3. Please compare the chances of developing lung diseases with other people of the same age and sex. Compared to others, my chances of developing lung problems are:
   - Much less than other people of my age and gender
   - A little less than other people of my age and gender
   - Almost like other people of my age and gender
   - A little more than other people of my age and gender
   - Much more than other people of my age and gender

4. Please compare your chances of having any serious health problems in the future with others of the same age and sex. Compared to other people of my age and sex, my chances of developing any serious health problems in the future are:
   - Much less than other people of my age and gender
   - A little less than other people of my age and gender
   - Almost like other people of my age and gender
   - A little more than other people of my age and gender
   - Much more than other people of my age and gender

5. Is there a chance that you will experience a serious health problem in the future?
   - Never
   - It is very unlikely
   - It is unlikely
   - Average probability
   - Perhaps
   - It is very likely
   - Definitely happens
6. What is the probability that you will have a serious health problem in the future?
   - Most likely
   - Unlikely

   A. What percentage?
7. You expressed your beliefs about how objectively likely it is that you have a serious health problem in the future, now please express the probability of a serious health problem in the future from 0 to 100.
8. During the past 7 days, how much have you thought about the possibility of developing a serious physical illness in the future (even briefly)?
9. How many times did you smoke or decide to smoke in the last week?
10. In what percentage of those cases did you think about the dangers of smoking for your health?
11. In how many cases did you not even think about the possibility of getting sick from it?
12. How dangerous is smoking for you?

    ○ Not dangerous at all ○ Moderately dangerous ○ Very dangerous

13. How dangerous is it for you to drink 5 or more alcoholic drinks in a row?

○ Not dangerous at all ○ Moderately dangerous ○ Very dangerous

14. How dangerous is it for you not to follow a healthy and low-fat diet?

○ Not dangerous at all ○ Moderately dangerous ○ Very dangerous

15. How dangerous is it for you to spend your time outside without protection from the sun (sunscreens, hats, etc.)?

○ Not dangerous at all ○ Moderately dangerous ○ Very dangerous

Attitude and Subjective Norms Questions

Please express your opinions about the following sentences, assuming you have a skin disease, by saying I strongly agree, I agree, I have no opinion, I disagree and I strongly disagree.

1 - I often think about my health condition.

○ I strongly agree ○ I agree ○ I have no opinion ○ I disagree ○ I strongly disagree

2- I suffer from not recognizing my health conditions.

○ I strongly agree ○ I agree ○ I have no opinion ○ I disagree ○ I strongly disagree

3- I think more about my health now than before the diagnosis.

○ I strongly agree ○ I agree ○ I have no opinion ○ I disagree ○ I strongly disagree

4 - Currently, I have no physical health concerns.

○ I strongly agree ○ I agree ○ I have no opinion ○ I disagree ○ I strongly disagree

5- I am not worried about my health conditions more than others do.

○ I strongly agree ○ I agree ○ I have no opinion ○ I disagree ○ I strongly disagree

6- I feel that I should be a little worried about my future health condition.

○ I strongly agree ○ I agree ○ I have no opinion ○ I disagree ○ I strongly disagree

7- When planning for the future, I consider my health status.

○ I strongly agree ○ I agree ○ I have no opinion ○ I disagree ○ I strongly disagree

8- Compared to others with skin disease, I feel less worried about my health conditions.

○ I strongly agree ○ I agree ○ I have no opinion ○ I disagree ○ I strongly disagree

9- My future health condition is not my main concern.

○ I strongly agree ○ I agree ○ I have no opinion ○ I disagree ○ I strongly disagree

10- I am sometimes worried about my physical condition.

○ I strongly agree ○ I agree ○ I have no opinion ○ I disagree ○ I strongly disagree

11. Just before my regular medical check-ups, my anxiety about my health increases.

○ I strongly agree ○ I agree ○ I have no opinion ○ I disagree ○ I strongly disagree

12-I don't currently think more about my health conditions compared to when I am diagnosed with a disease.

○ I strongly agree ○ I agree ○ I have no opinion ○ I disagree ○ I strongly disagree

13 - I am not worried by uncertainty of my health status.

○ I strongly agree ○ I agree ○ I have no opinion ○ I disagree ○ I strongly disagree

14 - I tend to be more confident about my health status.

○ I strongly agree ○ I agree ○ I have no opinion ○ I disagree ○ I strongly disagree

15 - I rarely think about my health condition.

○ I strongly agree ○ I agree ○ I have no opinion ○ I disagree ○ I strongly disagree

16 - Because of my physical health, my future is very important to me.

○ I strongly agree ○ I agree ○ I have no opinion ○ I disagree ○ I strongly disagree

17 - I am not worried about the recurrence of my disease.

○ I strongly agree ○ I agree ○ I have no opinion ○ I disagree ○ I strongly disagree

18 - When I think about my future health status, I feel anxious.

○ I strongly agree ○ I agree ○ I have no opinion ○ I disagree ○ I strongly disagree

19 - Even minor pains remind me of my illness.

○ I strongly agree ○ I agree ○ I have no opinion ○ I disagree ○ I strongly disagree

20 - I feel optimistic when I focus on my future.

○ I strongly agree ○ I agree ○ I have no opinion ○ I disagree ○ I strongly disagree

21- I am worried that the problems of my illness may not end.

○ I strongly agree ○ I agree ○ I have no opinion ○ I disagree ○ I strongly disagree

22- When I read articles about my illness, I don't feel worried about my future.

○ I strongly agree ○ I agree ○ I have no opinion ○ I disagree ○ I strongly disagree

Perceived Behavioral Control Questions

In the next group of questions, we are interested in your thoughts on the following behaviors. Thank you for your detailed answers...

1. I use sunscreen every day for six hours from 10 am to 4 pm (grading from 1 to 7)
    - Harmful 1 / 2 / 3 / 4 / 5 / 6 / 7 Useful
    - Bad 1 / 2 / 3 / 4 / 5 / 6 / 7 Good
    - Worthless 1 / 2 / 3 / 4 / 5 / 6 / 7 Valuable
    - With difficulty 1 / 2 / 3 / 4 / 5 / 6 / 7 with pleasure.

The next few questions are about what the important people in your life think you should and shouldn't do:

2. They think you should 1 / 2 / 3 / 4 / 5 / 6 / 7 should not wear hats, gloves, and sunglasses to protect yourself from the sun whenever you are outside (for the next 6 months).
3. Most of the important and close people in my life use hats, sunscreens, and protective clothing to protect themselves from the sunlight when spending time outside.

    Definitely true 1 / 2 / 3 / 4 / 5 / 6 / 7 definitely false

4. If I want, I can use sunscreens outdoors from 10:00 to 4:00 PM for the next six months.

    Definitely true 1 / 2 / 3 / 4 / 5 / 6 / 7 definitely false

5. How likely are you to use sunscreen every day when you are outside?

    Low probability 1 / 2 / 3 / 4 / 5 / 6 / 7 very high probability

6. I expect myself to use sunscreens every day I go out.

    100% 1 / 2 / 3 / 4 / 5 / 6 / 7 10%

7. Have you ever lay down in the sun for tanning from 10 am to 4 pm?
    - Yes
    - No (if no, refer to question 10)
8. How many days in the week do you lay under the sun to tan during the summer?
9. On a normal day, how many hours do you lay under the sunlight to tan?
10. When you are outside in the middle of the day, do you:

Behavioral Intention Questions

1. How often do you stay in the shade?

○ Always ○ Often ○ Sometimes ○ Rarely

2. How often do you wear a hat?

○ Always ○ Often ○ Sometimes ○ Rarely

3. How often do you wear protective clothing (i.e. long sleeves, pants, etc.)?

○ Always ○ Often ○ Sometimes ○ Rarely

4. How often do you wear sunglasses?

○ Always ○ Often ○ Sometimes ○ Rarely

5. How often do you use sunscreens?

○ Always ○ Often ○ Sometimes ○ Rarely

Skin Cancer Preventive Questions

6. How much sun protection factor (SPF) in lotions and sunscreens do you use the most?
7. Have you ever used solarium or tanning beds?
    - Yes
    - No
8. If yes, how many times in a regular month:
9. In the past 30 days, have you been exposed to the sun or tanning beds?
    - Most of the time with friends
    - Most of the time when I am alone
10. What percentage of your time do you spend tanning?

The next question is about sunburns (Even those that involve your skin being red for more than 12 hours).

11. Have you had a sunburn in the last 12 months?
    - Yes
    - No
12. If the redness of the skin for more than 12 hours is considered as a sunburn, how many times have you had a sunburn in the last 12 months?
13. Who in your family regularly protects their skin from the sun (including sitting in the shade, wearing hats, clothes, or glasses, using sunscreens with high SPF) (put an X in front of each one if it is positive.)

Family members	Do they use sunscreens?	Do they live in the same house with you?
Mother		
Father		
Grand mother		
Grand father		
Brother(s)		
Sister(s)		
Others (name)		

14. Who in your family regularly tans (whether outdoors in the sunlight or in tanning beds)

Family members	Do they tan regularly?	Do they live in the same house with you?
Mother		
Father		
Grand mother		
Grand father		
Brother(s)		
Sister(s)		
Others (name)		

15. How many of your 10 closest friends use sunscreens regularly?
16. How many of your 10 closest friends tan regularly?
17. Does your best friend use sunscreens regularly?
18. Does your best friend tan regularly?

Thank you for your time
